# Changes in the Response Properties of Inferior Colliculus Neurons Relating to Tinnitus

**DOI:** 10.3389/fneur.2014.00203

**Published:** 2014-10-09

**Authors:** Joel I. Berger, Ben Coomber, Tobias T. Wells, Mark N. Wallace, Alan R. Palmer

**Affiliations:** ^1^MRC Institute of Hearing Research, University Park, Nottingham, UK

**Keywords:** acoustic over-exposure, auditory, behavior, electrophysiology, gap detection, response types, tinnitus, tinnitus animal model

## Abstract

Tinnitus is often identified in animal models by using the gap prepulse inhibition of acoustic startle. Impaired gap detection following acoustic over-exposure (AOE) is thought to be caused by tinnitus “filling in” the gap, thus, reducing its salience. This presumably involves altered perception, and could conceivably be caused by changes at the level of the neocortex, i.e., cortical reorganization. Alternatively, reduced gap detection ability might reflect poorer temporal processing in the brainstem, caused by AOE; in which case, impaired gap detection would not be a reliable indicator of tinnitus. We tested the latter hypothesis by examining gap detection in inferior colliculus (IC) neurons following AOE. Seven of nine unilaterally noise-exposed guinea pigs exhibited behavioral evidence of tinnitus. In these tinnitus animals, neural gap detection thresholds (GDTs) in the IC significantly increased in response to broadband noise stimuli, but not to pure tones or narrow-band noise. In addition, when IC neurons were sub-divided according to temporal response profile (onset vs. sustained firing patterns), we found a significant increase in the proportion of onset-type responses after AOE. Importantly, however, GDTs were still considerably shorter than gap durations commonly used in objective behavioral tests for tinnitus. These data indicate that the neural changes observed in the IC are insufficient to explain deficits in behavioral gap detection that are commonly attributed to tinnitus. The subtle changes in IC neuron response profiles following AOE warrant further investigation.

## Introduction

Tinnitus is a phantom sound percept that affects 10–15% of the populations of industrialized countries (1). Tinnitus has a wide range of etiologies, but the most common cause is exposure to excessively loud or persistent noise; ototoxic drugs can also trigger the onset of tinnitus [for a recent review, see Ref. (2)].

Early research into the underlying pathological mechanisms of tinnitus, using animal models, was hampered by the lack of an objective means of identifying tinnitus. However, the development of behavioral tests has allowed tinnitus research to advance rapidly (3–6). The most commonly used behavioral model – gap prepulse inhibition of acoustic startle (GPIAS) – relies on measuring an innate reflex response to a startling stimulus. Briefly, a loud sound evokes an acoustic startle reflex. When the startling sound is preceded by a gap in and otherwise continuous background noise, the magnitude of the startle response is reduced in size, a phenomenon known as prepulse inhibition [PPI; (6, 7)]. The impairment of gap detection (and subsequent PPI deficits) observed following acoustic over-exposure (AOE) or salicylate administration is thought to be caused by tinnitus “filling in” the gap, thus, reducing its salience (7–13). Others have suggested that behavioral deficits may actually reflect a deficit in temporal processing associated with the AOE, rather than tinnitus *per se* (9, 14).

Psychophysical gap detection thresholds (GDTs) have previously been correlated with neural gap detection responses (15). As a result, if neural GDTs were increased beyond the duration of the gap used in the behavioral test, this would highlight the underlying neural basis of behavioral deficits. Clearly, if impaired gap detection is to be used as an indicator for behavioral evidence of tinnitus, it is important to establish whether gap detection performance in the brain is within normal limits after AOE, as measured *in vivo* in auditory neurons. While Walton et al. (16) found no differences in neural GDTs between mice carrying a deafness gene and normal-hearing controls – indicating that temporal processing was not affected in these genetically modified mice – Yin et al. (17) demonstrated neural GDT deficits in guinea pigs (GPs) with a high-frequency hearing loss following AOE. However, no study to date has attempted to quantify neural GDTs in animals with behavioral evidence of tinnitus.

Previous work has implicated the inferior colliculus (IC), a midbrain auditory structure, in contributing to the generation of this phantom percept following AOE (18–20). A number of changes have been demonstrated in the response properties of IC neurons that include increased spontaneous firing rates (18, 19), tonotopic reorganization [in some cases; (21); although less conclusively in others; (22)], and increased bursting activity (8, 18). Alterations in both GABAergic and glycinergic inhibitory neurotransmission have been shown in the IC [for a review, see Ref. (23)], providing a plausible mechanism by which functional changes – such as modified patterns of neuronal firing – might occur.

Here, we investigated whether behavioral gap detection deficits – commonly attributed to tinnitus – can be explained by impaired temporal processing in the IC. To this end, we measured neuronal GDTs in the IC of GPs exhibiting behavioral evidence of tinnitus following unilateral AOE, and compared these with AOE GPs without behavioral evidence of tinnitus and unexposed control GPs. Neuronal GDTs in the IC have previously been shown to be in good agreement with those seen behaviorally (24), most likely due to the IC being an almost obligatory relay for the convergence of ascending auditory information (25). Consequently, inferences into behavioral performance can be reliably made from neural GDTs recorded in this auditory structure. Ultimately, this will provide insight into the neural mechanisms behind behavioral gap detection deficits following AOE, which is essential for understanding the reliability of the tinnitus behavioral model.

## Materials and Methods

### Animals

All procedures were carried out in accordance with the European Communities Council Directive of 24 November 1986 (86/609/EEC) and with the approval of the Animal Welfare and Ethical Review Body at the University of Nottingham, UK. Experiments were conducted on 15 male and female pigmented GPs weighing 300–500 g at the onset of behavioral testing. GPs were bred in-house and group-housed on a 12:12 h light:dark cycle. Food and water were freely available.

### Behavioral measure of tinnitus

The behavioral method used to identify tinnitus in this study is based on a gap detection paradigm devised by Turner et al. (6) in which we measured flexion of the pinna, or the Preyer reflex (7). The magnitude of the Preyer reflex is calculated as pinna displacement under different acoustic conditions, and these measurements are used to quantify gap-induced PPI of the reflex. In animals experiencing tinnitus, PPI is compromised; hence, the paradigm allows objective identification of tinnitus. This method is described in detail elsewhere (7, 8).

### Baseline behavioral testing

Baseline PPI of the Preyer reflex was measured in each GP over a 2-week period (minimum of three and a maximum of six testing sessions). Startling stimuli [broadband noise (BBN) bursts of 20 ms; rise/fall time of 1 ms] and continuous background noise conditions [either BBN or 2 kHz wide narrow-band noise (NBN) centered at 5, 9, 13, or 17 kHz] were used, as described previously (7, 8). The gap used to elicit gap-induced PPI in the present study was 50 ms in duration, consistent with that used by others [e.g., Ref. (6, 12, 13)]. After 2 weeks of baseline data collection, the significance of PPI was calculated as described in Berger et al. (7). Briefly, baseline data were pooled and the differences between “no gap” and “gap” trials were tested for statistical significance using a Wilcoxon rank-sum test to a 95% confidence rating for each GP at each background frequency. GPs that exhibited significant PPI in all background sound conditions were unilaterally exposed to loud noise (*n* = 9).

### Acoustic over-exposure

Guinea pigs were anesthetized with ketamine (Ketaset; 50 mg kg^−1^, i.p.; Fort Dodge Animal Health Ltd., Southampton, UK) and xylazine (Rompun; 10 mg kg^−1^, i.p.; Bayer PLC, Newbury, UK), supplemented with further administrations of a mixture of ketamine and xylazine, in a ratio of 15:2 (i.m.), throughout the procedure. A homeothermic heating pad (Harvard Apparatus Ltd., Edenbridge, UK) and a rectal probe were used to monitor core body temperature and maintain it at 38 ± 0.5°C. Auditory brainstem responses (ABRs) were recorded prior to, immediately after, and 8 weeks following AOE to determine hearing thresholds, in the same manner as described previously (8). GPs were placed inside a sound-attenuated booth and remained there for the duration of the ABR recording and AOE. Following the collection of pre-trauma ABR thresholds, GPs were exposed to NBN bursts (duration of 500 ms and inter stimulus interval of 200 ms; center frequency 10 kHz; bandwidth 1 kHz), presented to the left ear only at 120 dB SPL, for 1 h via a 25 mm loud speaker (Peerless DX25, Tymphany, Hong Kong), connected to a 20 mm diameter polyethylene tube in order to form a seal around the ear and maintain a closed sound system. To minimize the risk of damage to the contralateral (right) ear, the right pinna was folded over and a polyethylene tube plugged with cotton wool was placed over the ear. ABRs recorded immediately after AOE confirmed that trauma occurred unilaterally. The AOE protocol was designed to minimize long-term hearing threshold shifts.

### Behavioral classification of tinnitus

We employed a commonly used classification for identifying behavioral evidence of tinnitus [e.g., Ref. (8, 10, 26, 27)]. Baseline gap detection data were pooled across all sessions. These were compared to pooled data acquired 7–8 weeks after AOE. This time-point was selected based on the assumption that tinnitus develops within 7–8 weeks following AOE (6, 13) and that hyperactivity becomes no longer dependent on cochlear input ~6 weeks after AOE (19, 20). PPI at the 7–8 week time-point was expressed as a ratio compared to baseline (before/after). Thus, a value of 1 indicated no change in PPI (relative to baseline), whereas a value <1 indicated a reduction in PPI, while a value >1 suggested that an improvement in PPI. Significant reductions in gap-induced PPI at any background frequency (compared to baseline) were identified using a two-way analysis of variance (ANOVA) with a Bonferroni *post hoc* test (*P* < 0.05). GPs that exhibited a significant reduction in gap-induced PPI at one or more background frequencies were categorized as “tinnitus” animals, while those that did not were categorized as “no tinnitus” animals.

### Surgery for neurophysiology

Following behavioral confirmation of tinnitus, GPs were anesthetized with urethane (0.5 g kg^−1^ in 20% solution, i.p.; Sigma, UK), ketamine (40 mg kg^−1^, i.p.), and xylazine (8 mg kg^−1^, i.p.), supplemented with further administrations of a mixture of ketamine and xylazine, in a ratio of 15:2 (i.m.), throughout the procedure to maintain areflexia. A single injection of atropine sulfate (0.06 mg kg^−1^, s.c.) was administered to suppress bronchial secretions. ABRs were then recorded to determine whether any permanent hearing threshold deficits were present. Following ABR recordings, GPs were tracheotomized and respired with 100% oxygen to maintain normal end-tidal CO_2_ partial pressure within a range of 28–38 mmHg. Core body temperature was monitored throughout, as described in the previous section. Animals were held in place using a stereotaxic frame, with hollow plastic speculae inserted into the ear canals replacing the ear bars. Throughout the duration of recording, animals remained inside a sound-attenuating chamber. Polyethylene cannulae (>10 cm length, 0.5 mm outer diameter) were inserted into the bullae on each side, in order to equalize pressure across the tympanic membrane while maintaining closed-field stimulation conditions. The posterior fossa was opened to reduce respiratory pulsations of the brain. Craniotomies were performed over the right and left IC (~4 mm diameter) using coordinates described in the guinea pig atlas of Rapisarda and Bacchelli (28). The Dura mater was excised and pairs of electrode arrays were slowly lowered into the brain at 10° to the vertical plane in the medial–lateral orientation. This was not only required to accommodate electrode arrays simultaneously on the two sides but also enabled a perpendicular approach to the isofrequency laminae of the IC (29). The exposed cortex was kept moist with intermittent application of warm 0.9% sodium chloride solution. When necessary, the brain surface was covered in 1.5% agar for stabilization during recording.

### Single-unit recording

To simultaneously record from left and right IC, two arrays of four glass-coated tungsten electrodes (~1–3 MΩ impedance) were attached to separate circuit boards (30). The tips of the electrodes were aligned and separated by ~200 μm. These electrode arrays were connected to a Tucker Davis Technologies (TDT) System 3 via TDT headstage amplifier and a TDT Medusa preamplifier. Electrodes were advanced into left and right IC, and extracellular single-units were recorded (filtered between 600 Hz and 3 kHz). Data were collected using Brainware (software developed by J. Schnupp, University of Oxford, UK).

### Auditory stimuli and recording procedure

Etymotic ER-4 earphones (Etymotic Research, Inc., IL, USA) were inserted into the hollow speculae, in order to present auditory stimuli diotically and create sealed acoustic systems. Search stimuli, generated using the TDT System 3, consisted of a wideband noise (duration 50 ms), with cosine-squared ramps lasting 8 ms and a repetition interval of 300 ms. Following online isolation of a single-unit, a frequency-response area was measured by presenting pure tone bursts (50 ms duration; 200 ms repetition interval) over a range of frequencies (50 Hz to ~25 kHz randomly interleaved at 0.25 octave intervals) and sound levels (attenuations of 0–95 dB in 5 dB steps, from a maximum of ~100 dB SPL). This enabled determination of the characteristic frequency (CF) of isolated single-units across multiple electrodes, which could potentially have disparate CFs.

### Neural gap detection thresholds

The minimum gap detection threshold (MGDT) was measured for each isolated single-unit in GPs with behaviorally identified tinnitus (*n* = 7), AOE GPs without tinnitus (*n* = 2) and in an additional group of control animals (*n* = 6) that were not noise-exposed. Auditory stimuli comprised a noise/tone burst (duration of 200 ms, on/off ramps of 0.5 ms), followed by a fixed-length period of silence or “gap” (durations of either 1, 2, 4, 8, 10, 20, 50 or 75 ms), and a final noise/tone burst (duration of 50 ms, on/off ramps of 2 ms). Each gap condition was presented in ascending order (20 repetitions, 700 ms repetition interval), for three types of carrier: (i) BBN, (ii) NBN (matched to the behavioral NBN background sound conditions, i.e., 4–6, 8–10, 12–14, or 16–18 kHz), or (iii) pure tones. Pure tones were presented at a frequency that matched the CF of each single-unit, as determined by frequency-response areas. NBN bursts were presented at the frequency range closest to the CF of a given neuron. In all cases, pre-gap and post-gap stimuli were spectrally identical, and were presented at the same sound level.

The sound level was determined based on that used in the behavioral test: as described in our previous work, we determined optimal sound levels of startling stimuli (95, 100, or 105 dB SPL) and background carrier (55, 60, or 70 dB SPL) to maximize baseline PPI for each animal [sound level-dependency test; see Ref. (7)]. In order to better compare behavioral performance and neural MGDTs (determined electrophysiologically), we selected analogous sound levels for both approaches. Although these were not directly comparable owing to a number of methodological differences (e.g., speaker type and anesthesia), this was deemed the most consistent way of selecting an appropriate sound level for MGDT measurement.

### Data analysis for MGDTs

Minimum gap detection thresholds of single-units were defined as the minimum gap duration where a significant increase in firing (>2SD above the mean firing rate within the preceding 50 ms) could be detected following the onset of the post-gap stimulus, i.e., a comparison of firing immediately after the gap vs. during or before the gap. These responses were further required to contain a minimum of three spikes (collected over 20 sweeps). MGDTs were assessed using custom-written Matlab software (R2009b, Math-Works, MA, USA). In addition, post-stimulus time histogram (PSTH) plots for each cell were visually inspected for confirmation purposes.

Single-unit MGDTs were calculated for each sound condition: pure tones (at CF), BBN, and NBN. For the NBN condition, single-units were included if the CF of a given single-unit was ≤1 kHz from the lower or upper limits of an NBN frequency range, e.g., single-units with a CF of 3–7 kHz were assessed in response to 4–6 kHz NBN stimuli. This was done to restrict the effect that having a NBN frequency substantially different from the CF may have on MGDTs. A small subset of units exhibited offset responses only, i.e., neuronal firing was suppressed during the presentation of auditory stimuli. For these single-units, MGDTs were determined solely by visual inspection, using a discernible increase in firing following the offset of the gap as an indication that the gap was detected.

The number of responsive neurons to each gap condition was expressed as a percentage of all recorded neurons in tinnitus and control groups. Mean MGDTs were also compared for tinnitus, no tinnitus, and control GPs for each noise condition, and differences between the three experimental groups statistically assessed with a Kruskal–Wallis test with a Dunn’s *post hoc* test. Data were initially pooled regardless of the side from which they were recorded, as we have previously demonstrated that a unilateral noise exposure resulted in bilateral increases in spontaneous firing rates (8) and this method of analysis preserved larger sample sizes. Analysis of MGDTs across left (ipsilateral) and right (contralateral) IC is further supported by the fact that the behavioral task involves binaural presentation of stimuli. However, MGDT data were subsequently analyzed independently for each side, as the unilateral AOE may have feasibly resulted in differences between sides. Statistical comparisons were made with a two-way ANOVA and Tukey’s HSD *post hoc* test.

### Response properties of IC neurons

Inferior colliculus single-units were sub-divided according to response profiles exhibited to gap detection stimuli. Three different classes were extracted from these data: (i) if a single-unit responded to the onset of the first 200 ms stimulus, but ceased firing within ~30 ms, it was labeled as an *onset* response; (ii) if a single-unit showed a response that lasted more than 30 ms of the first 200 ms stimulus, it was designated as a *sustained* response; (iii) single-units that were predominantly silent throughout the presentation of both the initial 200 ms stimulus and the second 50 ms stimulus, yet responded following the offset of either stimulus, were categorized as exhibiting an *offset* response. The classifications for onset and sustained-response single-units were based on the scheme of Astl et al. (31), while offset responses were classified in a similar manner to Kasai et al. (32).

The proportion of response types was calculated for control, no tinnitus, and tinnitus groups, and compared. Chi-squared tests were used to determine whether there were any significant differences in the distributions of response types overall or when sub-divided according to side. MGDT data were also sub-divided, according to response profile (and experimental group) and statistically assessed with a two-way ANOVA.

## Results

**Figure 1:**
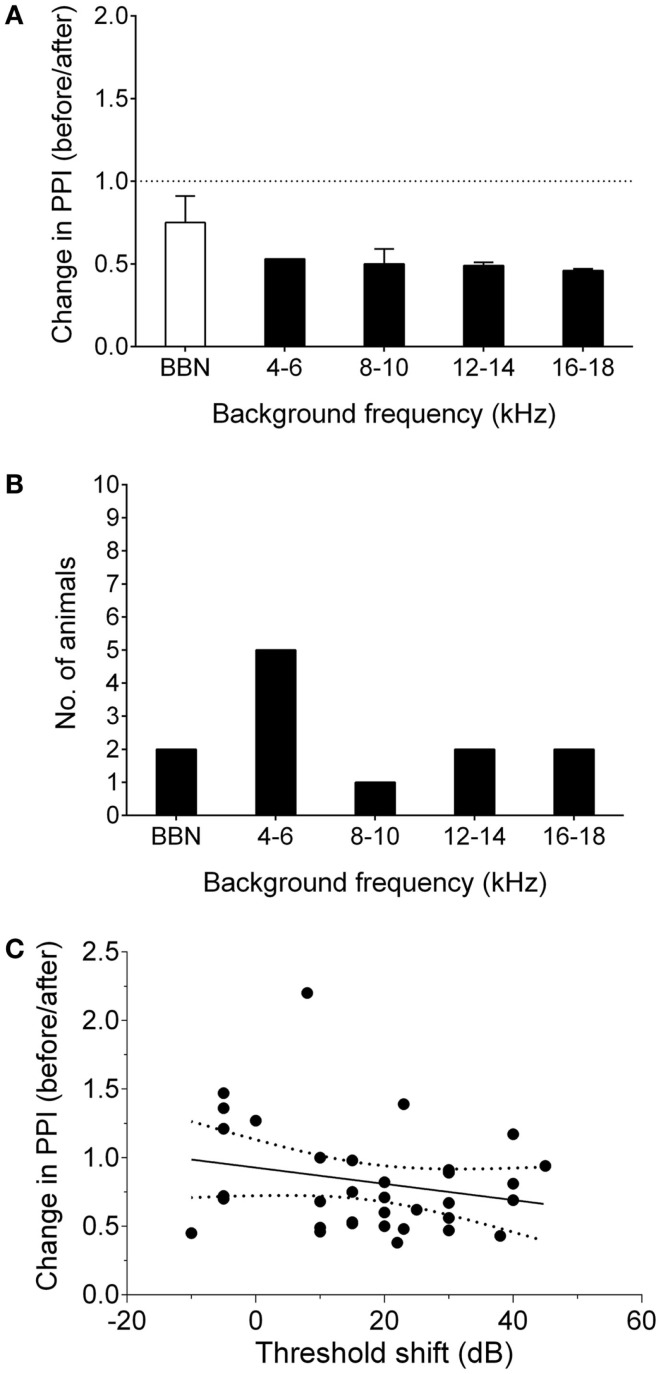
**Behavioral signs of tinnitus**. **(A)** Behavioral performance after AOE is shown for a representative “tinnitus” animal, expressed as change in PPI, i.e., a ratio of performance before vs. after noise exposure, at each background frequency. Values <1 indicate poorer gap detection, while a value close to 1 indicates no effect of noise exposure. Black bars indicate frequencies with significantly worse gap detection (*P* < 0.05). **(B)** The number of animals exhibiting gap detection deficits at each background frequency. Note: some animals exhibited gap detection deficits at more than one frequency. **(C)** There was no significant correlation between behavioral performance and ABR threshold shifts. Solid line indicated linear regression. Dotted lines show 95% confidence intervals. Each point represents the behavioral performance for a given background condition in a single animal, relative to the ABR threshold shift at the corresponding frequency.

Nine GPs were noise-exposed and tested for behavioral evidence of tinnitus 7–8 weeks following AOE. A further six animals were used solely for neurophysiological recordings, in order to serve as a control group (i.e., no noise exposure). An example of behavioral gap detection deficits in a tinnitus GP is shown in Figure 1A. Seven of the nine noise-exposed GPs exhibited behavioral evidence of tinnitus. This equates to ~75% of animals developing tinnitus, which is consistent with data from our previous study using the same AOE paradigm and behavioral criteria (8). Across animals, 4–6 kHz was the most common background sound frequency at which significant gap detection deficits were found, although some GPs showed a significant decrease in behavioral gap detection at multiple frequencies following AOE (Figure 1).

Linear regression analysis comparing ABR threshold shift with change in PPI (for each background frequency) was used to determine whether the behavioral gap detection deficits could simply be a result of hearing loss caused by AOE, i.e., whether tinnitus-like behavior worsened with increasing hearing threshold shifts. There was no significant correlation between ABR threshold shifts and PPI deficits (*r* = 0.05; *P* = 0.18), suggesting that the behavioral gap detection deficits observed here were not simply a result of reduced hearing thresholds (Figure 1C).

In our data, behavioral gap detection deficits were not restricted to the noise exposure frequency. This is in contrast with previous animal studies using the gap detection method, e.g., in mice (11) and gerbils (33). The variability in tinnitus frequency in the present study can, to some extent, be explained by the noise exposure paradigm. Despite using NBN exposure, broadband shifts in ABR thresholds on the exposed side were observed (18.57 dB HL, ±6.14 for 5 kHz, 16.43 dB HL, ±4.72 for 10 kHz, and 18.57 dB HL, ±6.96 for 15 kHz), which is consistent with our previous data (8). This is in keeping with the findings of some other groups (9, 34). Therefore, given that the immediate effects were broadband, any damage that resulted in tinnitus may not have been restricted to one particular frequency. Behavioral deficits not restricted to the noise exposure frequency have also been demonstrated previously in rats (34, 35), and Engineer et al. (36) demonstrated behavioral deficits centered below the noise exposure frequency. Moreover, human studies have demonstrated considerable variability in frequency estimates of tinnitus (37–40).

### Changes in IC single-unit response types following AOE

**Figure 2:**
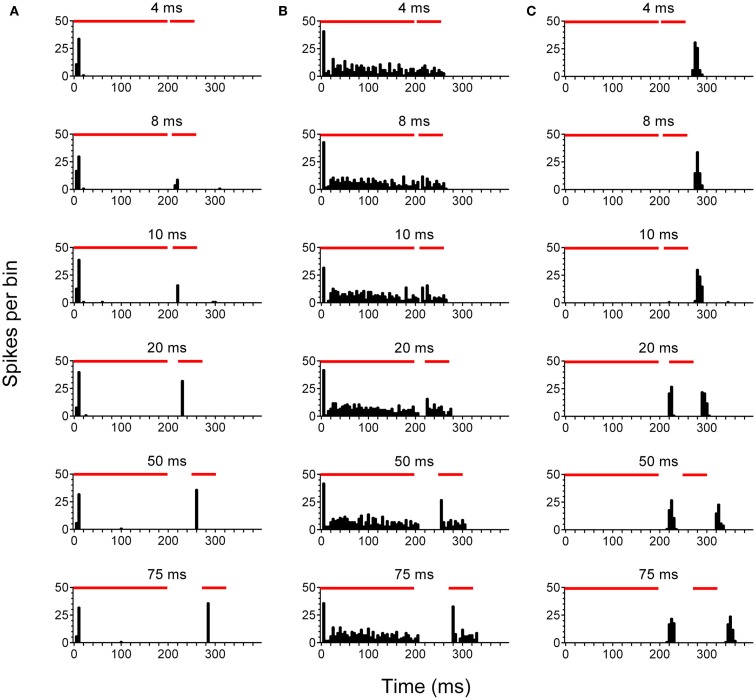
**Neuronal activity in the inferior colliculus**. Representative examples are shown of onset **(A)**, sustained **(B)**, and offset **(C)** single-units recorded in the IC. PSTH plots were generated in response to pure tone gap detection stimuli incorporating gaps of varying duration 4, 8, 10, 20, 50, and 75 ms are shown. Red bars indicate the duration of the first 200 ms stimulus, followed by a variable gap, and finally the second 50 ms stimulus.

**Figure 3:**
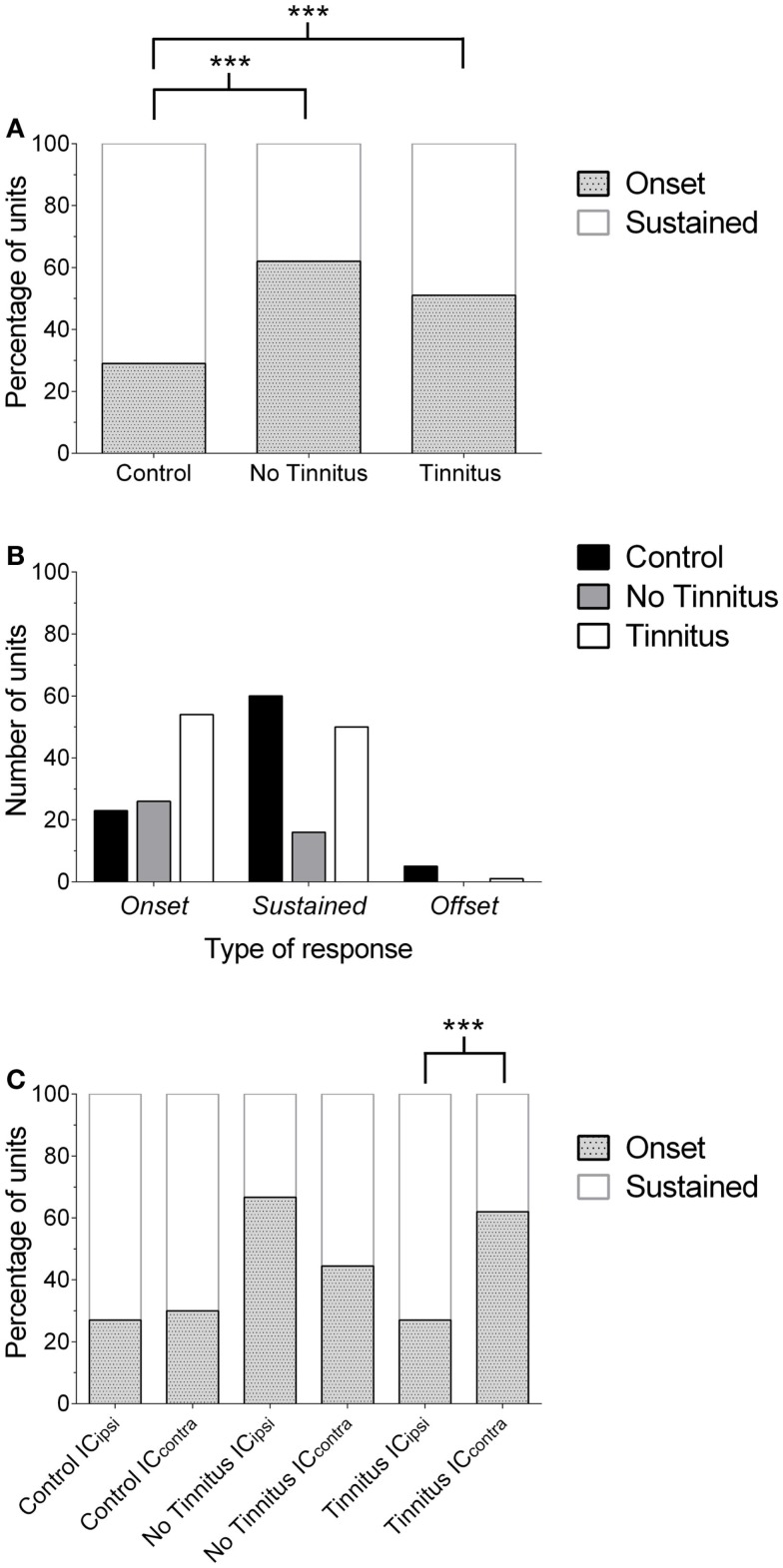
**Changes in the proportion of IC response profiles**. **(A)** The proportion of single-units exhibiting either an onset or sustained-response profile is shown for each experimental group. Offset units have been excluded from this graph for the purposes of clarity (due to their low incidence). Onset responses significantly increased (relative to sustained units) in no tinnitus and tinnitus animals, compared with controls (***P < 0.0001). **(B)** Number of units recorded for each experimental group, separated according to response-type classification. The number of units recorded in no tinnitus GPs was considerably lower than controls and tinnitus GPs. **(C)** The increase in the proportion of onset units in tinnitus GPs was isolated to the contralateral (relative to AOE) IC (***P < 0.0001). In no tinnitus GPs, this increase was present in both the IC_ipsi_ and the IC_contra_.

After concluding behavioral testing, single-unit recordings were performed in the left, ipsilateral IC (IC_ipsi_) and right, contralateral IC (IC_contra_) of control (*n* = 88 cells), no tinnitus (*n* = 42 cells), and tinnitus (*n* = 105 cells) groups of GPs. Figure 2 highlights the three categories under which IC units were classified, and also shows responses to pure tone stimuli with varying gap lengths, as used for determining MGDTs: Figure 2A shows a representative example of a cell responding to the stimulus onset; Figure 2B shows a cell exhibiting a sustained response; Figure 2C shows a cell responding only to the offset of a stimulus. Data from no tinnitus, tinnitus, and control groups were analyzed to determine whether there were any changes in proportions of the types of responses exhibited by IC neurons. The percentage of “onset” and “sustained” single-unit types in response to pure tone stimuli for each group are shown in Figure 3A.

In controls, 26% of cells exhibited onset responses (*n* = 23), 68% were of a sustained type (*n* = 60), and 6% displayed offset responses (*n* = 5). In tinnitus animals, 51% of units were onset-responders (*n* = 54), while 48% of units were classified as sustained responses (*n* = 50), and only 1% of units demonstrated offset responses (*n* = 1). In no tinnitus animals, 62% of units were onset-responders (*n* = 26), while 38% of units were classified as sustained responses (*n* = 16). Chi-squared tests were applied to compare the frequency with which different types of units occurred (excluding offset responses due to the extremely low incidence), this revealed that tinnitus GPs – proportionally – exhibited significantly fewer sustained responses than controls and significantly more onset responses [χ^2^(1) = 29.52, *P* < 0.0001]. When comparing no tinnitus GPs to controls, there were again significantly fewer sustained responses and significantly more onset responses [χ^2^(1) = 23.95, *P* < 0.0001]. There was no significant difference between no tinnitus and tinnitus GPs in response types [χ^2^(1) = 1.65, *P* = 0.20]. However, as Figure 3B highlights, overall there were considerably fewer recorded units in no tinnitus GPs (compared to the other two groups) meaning that the sample size was not large enough to determine whether changes in this group were reliable or a result of a sampling bias.

In order to determine whether the change in the proportion of response types between control, no tinnitus, and tinnitus groups was limited to a specific side, data were analyzed for IC_ipsi_ and IC_contra_ separately (Figure 3C). Offset units were again excluded from analysis due to their low incidence (*n* = 6 across all experimental groups). In control animals, there was no significant difference between the two sides: 27% of units recorded from the IC_ipsi_ exhibited onset responses (*n* = 15) and 73% exhibited sustained responses (*n* = 41), compared with 30% onset (*n* = 8), and 70% sustained (*n* = 19) from the IC_contra_ [χ^2^(1) = 0.10, *P* = 0.76]. In tinnitus animals, separating data according to side clearly highlighted where response-type differences between the two experimental groups occurred: the proportions of response types recorded from the IC_ipsi_ were similar to controls; 27% onset responses (*n* = 8) and 73% sustained responses (*n* = 22). However, in the IC_contra_ of tinnitus GPs, 62% of units exhibited onset responses (*n* = 46), compared with only 38% sustained responses (*n* = 28). The difference in the proportion of onset vs. sustained response types between IC_ipsi_ and IC_contra_ in tinnitus GPs was highly significant [χ^2^(1) = 46.42, *P* < 0.0001]. In no tinnitus GPs, the change in the proportion of onset types was evident for both the IC_ipsi_ and the IC_contra_, and there was no significant difference between the two sides: 67% of units recorded from the IC_ipsi_ exhibited onset responses (*n* = 22) and 33% exhibited sustained responses (*n* = 11), compared with 44% onset (*n* = 4), and 56% sustained (*n* = 5) from the IC_contra_ [χ^2^(1) = 2.07, *P* = 0.15]. It should be noted, however, that the sample sizes in the IC_contra_ in this group were very small when separated in this manner.

In summary, AOE resulted in a marked shift in IC single-unit response types, i.e., a significantly higher proportion of units classified as onset-responders, compared with controls. In tinnitus animals, this shift was only found in the IC_contra_, which – taken alone – is perhaps not altogether surprising given the unilateral nature of the AOE paradigm. In no tinnitus animals, this shift was evident to a greater degree in IC_ipsi_ compared with IC_contra_, although there was no significant difference between the two sides. The mechanisms underlying the difference in the laterality of effects between no tinnitus and tinnitus GPs are unclear, and are difficult to resolve with the present data.

### Neural gap detection thresholds with pure tone stimuli

**Figure 4:**
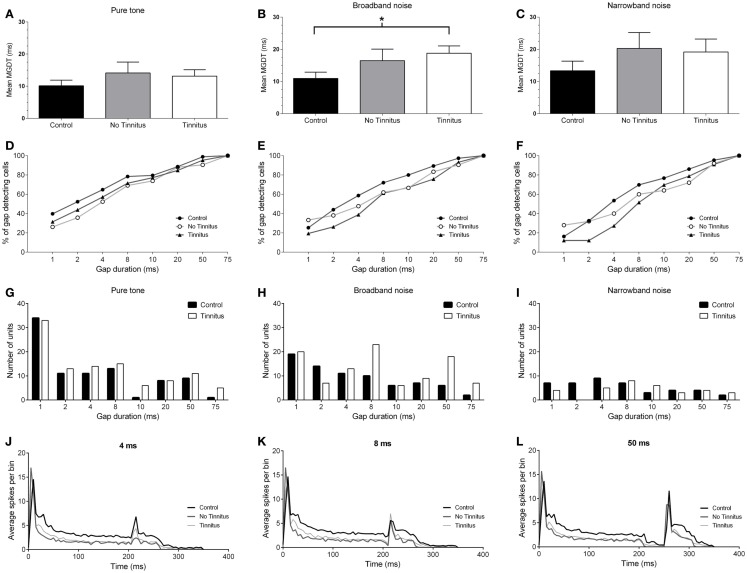
**Neural gap detection in the IC**. **(A)** Mean MGDTs in response to pure tone stimuli at CF for control (*n* = 96), no tinnitus (*n* = 42), and tinnitus (*n* = 109) animals. **(B)** Mean MGDTs in response to BBN for control (*n* = 76), no tinnitus (*n* = 42), and tinnitus (*n* = 103) groups (*P < 0.05). **(C)** Mean MGDTs in response to NBN for control (*n* = 43), no tinnitus (*n* = 25), and tinnitus (*n* = 33) GPs. **(D)** The percentage of gap-detecting cells, relative to total cell count in response to pure tone stimuli for control, no tinnitus, and tinnitus GPs. For each gap duration (1, 2, 4, 8, 10, 20, 50, and 75 ms) the percentage of cells able to detect a gap less than or equal to a given duration is shown. **(E)** The percentage of gap-detecting cells with a BBN carrier. **(F)** The percentage of gap-detecting cells with an NBN carrier. **(G)** Distribution of the number of units with MGDTs for each gap duration in response to pure tone stimuli, shown for control and tinnitus GPs. For the purposes of clarity, no tinnitus GPs have been omitted. **(H)** Distribution of MGDTs in response to BBN stimuli. **(I)** Distribution of MGDTs in response to NBN stimuli. **(J)** The averaged population responses of IC neurons in control (black line), no tinnitus (dark gray line), and tinnitus (light gray line) groups for gaps of 4 ms duration, in response to a pure tone carrier. **(K)** Averaged population responses to gaps of 8 ms duration. **(L)** Averaged population responses to gaps of 50 ms duration.

The mean (±SEM) MGDTs for pure tone stimuli, separated according to experimental group, are shown in Figure 4A. MGDTs were 10.22 ms (±1.75 ms; *n* = 88) for controls, 14.12 ms (±3.39 ms; *n* = 42) for no tinnitus GPs, and 13.14 ms (±1.97 ms; *n* = 105) for tinnitus GPs; no statistically significant differences were found between the three groups (*P* = 0.09). Pure tone MGDTs were also plotted as a percentage of the total number of cells responding to each gap duration tested in Figure 4D. In control GPs, 99% of single-units exhibited MGDTs of 50 ms or less, while in no tinnitus GPs, MGDTs of 50 ms or less were observed in 90% of cells and in tinnitus GPs, MGDTs of 50 ms or less were observed in 95% of cells recorded.

### Neural gap detection in response to a BBN stimulus

Figure 4B shows the mean MGDTs, and Figure 4E shows the percentage of gap-detecting cells for each group in response to BBN. Mean (±SEM) MGDTs in response to BBN were 10.95 ms (±1.97 ms; *n* = 75) for controls, 16.48 ms (±3.58 ms; *n* = 42) for no tinnitus GPs, and 18.79 ms (±2.27 ms; *n* = 103) for tinnitus GPs. MGDTs were significantly longer for tinnitus GPs compared with controls (*P* < 0.05), but not when compared with no tinnitus GPs. However, importantly, the percentage of units with MGDTs of 50 ms or less were similar for control (97%), no tinnitus (92%), and tinnitus GPs (93%). The sample size for responses to BBN and NBN stimuli was smaller than for pure tones, as in some cases cells either did not respond to these stimuli, or exhibited a CF >1 kHz from the NBN frequency range.

### Gap detection in response to NBN stimuli

Figure 4C shows the mean MGDTs for NBN, while the percentage of gap-detecting cells are shown in Figure 4F. Mean (±SEM) MGDTs were 13.33 ms (±3.64 ms; *n* = 43) for controls, 20.28 ms (±4.96 ms; *n* = 25) for no tinnitus GPs, and 19.18 ms (±4.03 ms; *n* = 33) for tinnitus GPs. No significant differences were evident between the three groups (*P* = 0.18) and, once again, the percentage of units with MGDTs ≤50 ms were similar for control (95%), no tinnitus (92%), and tinnitus (91%) groups.

To summarize, although MGDTs were significantly increased in tinnitus GPs in response to BBN stimuli, the effects on the percentage of cells capable of detecting gaps of ≤50 ms were negligible, regardless of the characteristics of the stimulus or the experimental group. A lack of dramatic change in neural gap detection in tinnitus GPs is further highlighted by Figures 4G–I, which show the number of units with MGDTs at each gap duration in response to the three different stimulus conditions. For the purposes of clarity, no tinnitus GPs have been omitted from these histograms. These figures clearly demonstrate that many units responded to gaps of short durations, regardless of whether they were recorded from tinnitus GPs or not.

### Population responses to gaps

Figures 4J–L show the averaged population responses for each experimental group to gaps with durations of 4, 8, and 50 ms, respectively, using a pure tone carrier. Although the response to the second stimulus onset was weaker in tinnitus GPs for the 4 ms gap condition (Figure 4J) – indicating slightly poorer gap detection – the response to the second stimulus with an 8 ms gap in tinnitus GPs actually slightly exceeded that of control GPs (Figure 4K), and control and tinnitus GPs were equivalent when a 50 ms gap was presented (Figure 4L). This further highlights that, while some minor differences were evident for short-gap durations, the detectability of gaps ≥8 ms was similar for controls and tinnitus GPs. For no tinnitus GPs, responses to 4 ms gaps were clearly reduced compared to both control GPs and tinnitus GPs. For gaps ≥8 ms duration, responses were similar to those of controls. The apparent reduction in sustained firing (following the onset response) in tinnitus and no tinnitus GPs was most likely a result of the proportional reduction in sustained single-unit responses in these groups.

### Laterality in neural gap detection

**Figure 5:**
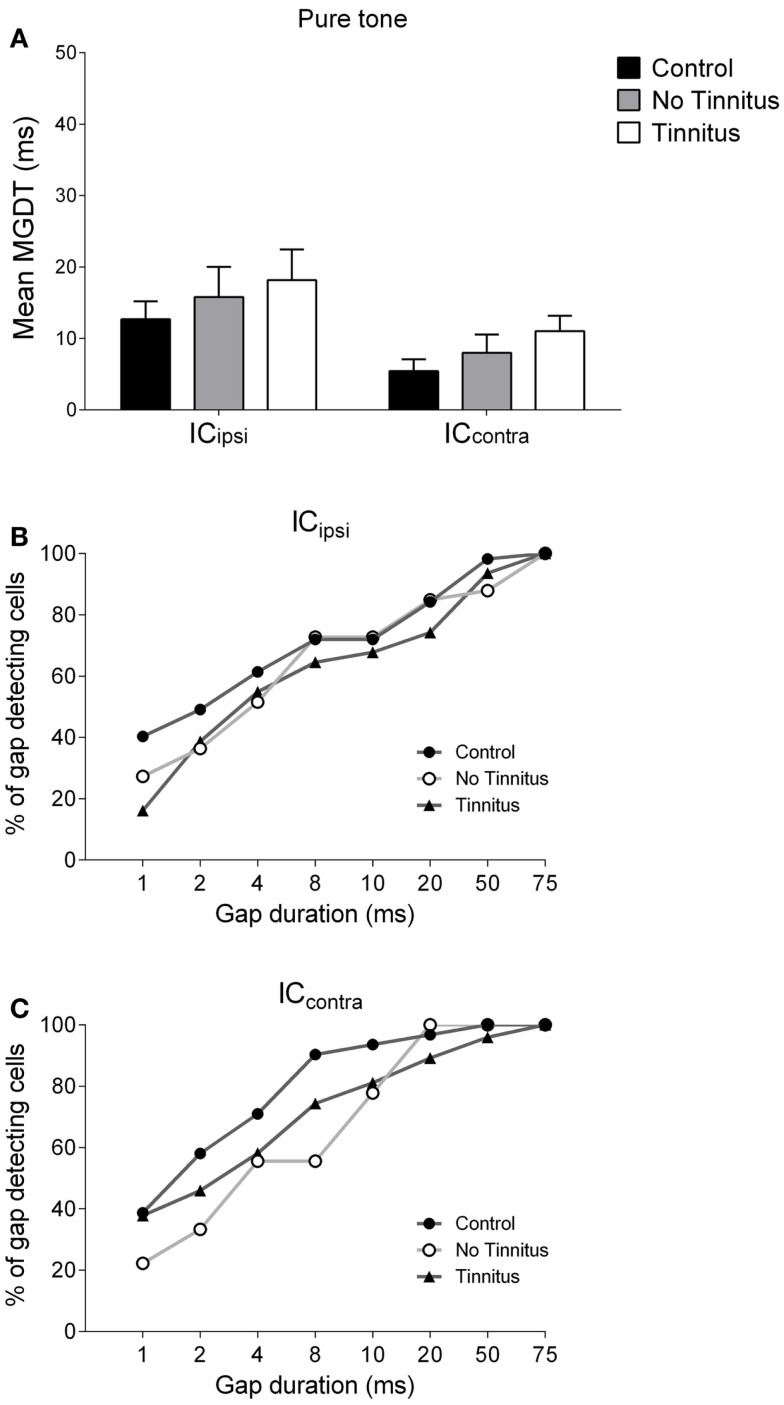
**Hemispheric differences in gap detection for pure tones**. **(A)** Mean MGDTs in response to pure tones are shown when data were sub-divided according to recording side. The percentage of gap-detecting cells are also shown for left IC **(B)** and right IC **(C)** in response to pure tones.

**Figure 6:**
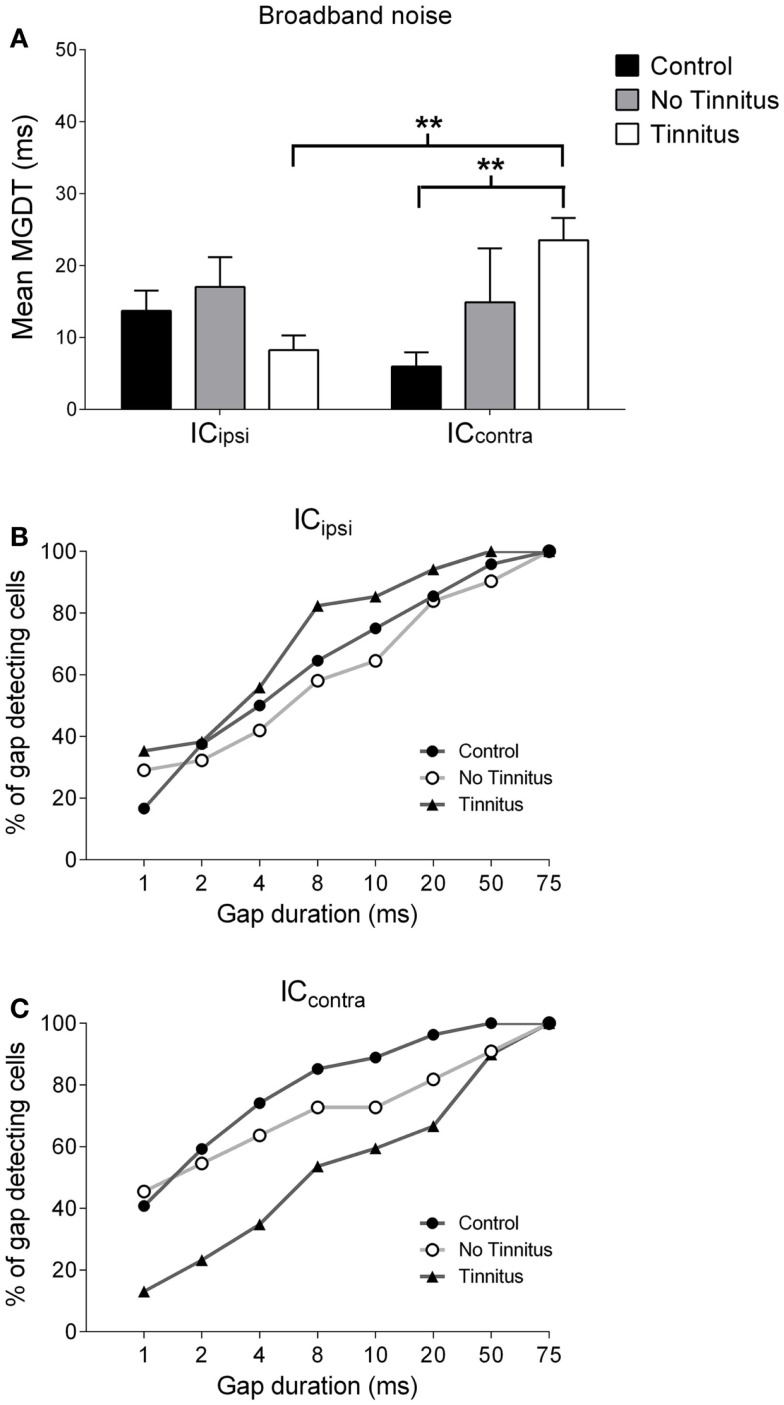
**Hemispheric differences in gap detection for BBN**. **(A)** Mean MGDTs with a BBN carrier from control, no tinnitus, and tinnitus animals, sub-divided according to recording side (**P < 0.01). The percentage of gap-detecting cells for left IC **(B)** and right IC **(C)** are also shown.

Due to the unilateral nature of the AOE protocol, it was entirely plausible that any changes in MGDTs may have been restricted solely to the IC_contra_. Thus, pooling data from both sides may have diluted any AOE-related changes in MGDTs. Consequently, MGDTs in response to pure tones and BBN were sub-divided according to recording side, for control, no tinnitus, and tinnitus GPs (Figures 5 and 6). This was not feasible for NBN data owing to the small sample size.

Figure 5A shows mean MGDTs (±SEM) in response to pure tones for control, no tinnitus, and tinnitus GPs, separated for IC_ipsi_ and IC_contra_. The percentage of gap-detecting cells in IC_ipsi_ and IC_contra_ are shown in Figures 5B,C, respectively. Observationally, the IC_contra_ exhibited shorter MGDTs than the IC_ipsi_ in controls, no tinnitus, and tinnitus groups, indicating a right-side dominant asymmetry. Statistical analysis revealed a significantly shorter MGDT overall in the IC_contra_ compared with the IC_ipsi_ [*F*_(1, 229)_ = 5.72; *P* < 0.05], but no significant effect of experimental group [*F*_(2, 229)_ = 1.07, *P* = 0.35], and no collicular side × group interaction [*F*_(2, 229)_ = 0.004, *P* = 0.99]. *Post hoc* analyses, comparing sub-divided IC_ipsi_ control (*n* = 57), IC_contra_ control (*n* = 31), IC_ipsi_ no tinnitus (*n* = 33), IC_contra_ no tinnitus (*n* = 9), IC_ipsi_ tinnitus (*n* = 31), and IC_contra_ tinnitus (*n* = 74) recordings, revealed no further differences either as a “within-group” comparison (i.e., IC_ipsi_ vs. IC_contra_) or “between-group” comparison (e.g., control IC_ipsi_ vs. tinnitus IC_ipsi_). The percentage of cells with MGDTs of ≤50 ms were 98% (IC_ipsi_) and 100% (IC_contra_) for control GPs, while for tinnitus GPs these numbers were slightly lower at 94% (IC_ipsi_) and 96% (IC_contra_). In no tinnitus GPs, these numbers were lower for the IC_ipsi_ (88%) and the same as controls for the IC_contra_ (100%).

Figures 6A–C show mean MGDTs and the percentage of gap-detecting cells in response to a BBN stimulus, separated according to side, for control, no tinnitus, and tinnitus GPs. Overall, there were no significant differences between the two sides [*F*_(1, 223)_ = 0.30; *P* = 0.58], nor across experimental groups [*F*_(2, 223)_ = 1.52; *P* = 0.22], but a group × side interaction was present [*F*_(2, 223)_ = 4.43; *P* < 0.05]. *Post hoc* analyses indicated that the IC_contra_ in tinnitus GPs (*n* = 74) had significantly longer MGDTs than both the IC_ipsi_ in tinnitus GPs (the “within-group” comparison; *n* = 34, *P* < 0.01) and the IC_contra_ in control GPs (the “between-group” comparison; *n* = 31, *P* < 0.01). Thus, neural GDTs to BBN stimuli were significantly worse in IC_contra_ of tinnitus GPs, which reflects activity generated by the AOE-treated ear. In response to BBN, the percentage of cells with MGDTs of 50 ms or less was 96% (IC_ipsi_) and 100% (IC_contra_) for control GPs. For tinnitus GPs, the percentage of units with thresholds of ≤50 ms was higher than controls for the IC_ipsi_ (100%) but lower for the IC_contra_ (90%). This contrasts with responses to pure tone stimuli, i.e., no change in the trend toward right-side-dominance, simply a small increase in MGDT relative to controls. For no tinnitus GPs, the percentage of units with thresholds of ≤50 ms was 90% (IC_ipsi_) and 91% (IC_contra_), but the small sample sizes in this group (relative to control and tinnitus GPs) should be noted.

### Neural gap detection in onset and sustained-response single-units

Next, onset and sustained units were compared to establish whether the changes in neural gap detection were more pronounced in either of these sub-classes. Across the three experimental groups (pooled across both recording sides), in response to the pure tone stimulus, onset cells displayed a mean (±SEM) MGDT of 15.74 ms (±2.16 ms; *n* = 103). The mean MGDT for sustained units was considerably shorter at 7.45 ms (±1.43 ms; *n* = 126). Offset units were excluded from analysis due to their low incidence in the sample (*n* = 6 across both groups), although the mean MGDT of offset units was considerably longer at 30.5 ms (±10.81 ms; *n* = 6), this is consistent with the results of other previous studies (16, 41).

**Figure 7:**
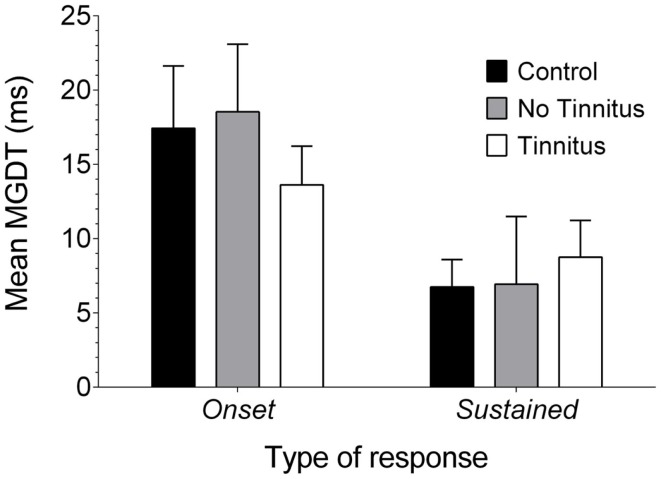
**Neural GDTs in the different types of IC neurons**. Mean MGDTs are shown for onset and sustained units (offset units were excluded owing to a small sample size) in control, no tinnitus, and tinnitus GPs.

Data were also examined to see whether MGDTs (in response to pure tones, as this was the largest sample available) varied as a function of response type for each experimental group (Figure 7): the mean (±SEM) MGDT in control GPs for onset units was 17.43 ms (±4.19 ms; *n* = 23), while sustained units had a mean MGDT of 6.75 ms (±1.85 ms; *n* = 56). In no tinnitus GPs, mean MDGTs were 18.54 ms (±4.55 ms; *n* = 26) for onset units and 6.94 ms (±4.56 ms; *n* = 16) for sustained units. Mean MDGTs in tinnitus GPs were 13.62 ms (±2.66 ms; *n* = 53) for onset units and 8.76 ms (±2.42 ms; *n* = 50) for sustained units.

Statistical analysis indicated a significant overall effect of response type on MGDT [*F*_(1, 218)_ = 11.15, *P* < 0.01]. However, there were no significant differences between experimental groups [*F*_(2, 218)_ = 0.11, *P* = 0.90], nor was there an interaction between experimental group and response type [*F*_(2, 218)_ = 0.61; *P* = 0.55] or any *post hoc* differences. In other words, sustained units had significantly shorter MGDTs than onset units, irrespective of experimental group.

## Discussion

The present study examined the effects of AOE, and the subsequent development of tinnitus, on neural gap detection in the IC. Behavioral evidence of tinnitus was demonstrated in ~75% of animals 7–8 weeks after AOE by showing a significant reduction in their ability to detect a 50 ms gap. There was a notable shift in the single-unit response types in the IC_contra_ (reflecting a left-side unilateral exposure) of tinnitus GPs, with proportionally more units classified as onset-responders, compared with control GPs. In no tinnitus GPs, a similar shift in response types was observed in both the IC_ipsi_ and the IC_contra_, although the sample sizes in this experimental group were relatively small. While some significant increases in neural GDTs were found in animals with tinnitus, it was clear that a 50 ms gap was detectable by the majority of IC neurons, regardless of whether or not a GP was subjected to AOE. Also, the distribution of MGDTs indicated that a large number of neurons still responded to gaps of 1 ms duration in animals after AOE. Moreover, in response to pure tones, MGDTs were considerably shorter in the IC_contra_ (right side) compared with the IC_ipsi_ (left side) – regardless of whether animals were noise-exposed or not – suggesting that a possible right-side advantage in temporal processing in this species.

Previously, Walton et al. (16) demonstrated that there were no significant differences in MGDTs between middle-aged mice that were genetically predisposed to develop moderate hearing loss and normal-hearing young mice. This contrasts with psychophysical evidence, showing that GDTs are significantly longer in hearing-impaired human beings when sensation levels are matched to a normal-hearing population, even at a young age (42).

The present study showed that while some deficits in neural gap detection were present following AOE, these were not evident at 50 ms gap duration, as used in the behavioral test. These data add to an already considerable body of evidence examining factors that affect gap detection. It is important to highlight that the estimates of MGDTs found here are considerably longer than those of Walton et al. (16, 43). However, in their studies, sound levels were matched to the best response of each single-unit. In the present study, in order to best model the conditions of the behavioral tinnitus test, sound levels used in the neural gap detection experiments were matched with those from the behavioral test. As a result, the levels were not necessarily optimal for each unit; hence, the estimates of MGDTs may have been longer. Furthermore, the MGDT estimates presented here are very similar to the psychophysically estimated thresholds of Fitzgibbons and Wightman (42).

Given that changes in sensitivity to a 50 ms gap were negligible in both our tinnitus and no tinnitus animals, as well as the fact that a similar number of units responded to gaps of very short durations compared with unexposed controls, deficits in neural gap detection ability at the level of the IC following AOE are unlikely to explain behavioral gap detection deficits. However, there is an important limitation of the present study. Since most of the animals only demonstrated deficits in behavioral gap detection at particular NBN frequencies, it is reasonable to predict that neural gap detection deficits, if they were present, would be most likely to occur in neurons that responded preferentially to these frequencies. Unfortunately, in the present study, this comparison was not possible owing to a small sample size of units with CFs falling within the NBN frequency where behavioral gap detection deficits occurred. This was a result of the recording procedure which, by starting in dorsal IC, skewed our data toward low frequencies. Consequently, future experiments should examine neural GDTs primarily at frequencies where behavioral gap detection deficits are present, as this would provide further insight into the underlying mechanisms of such deficits. Furthermore, it would be of considerable benefit to determine whether any gap detection deficits were evident in the auditory cortex.

Due to the nature of our stimuli, i.e., RMS sound levels for pure tone, NBN, and BBN were the same, it is likely that a reduction in the signal energy at the CF of a neuron would cause the sound level of the BBN stimulus to be lower at a given frequency by a considerable margin, relative to pure tone stimuli of the same frequency. Small AOE-related effects on neural GDTs in tinnitus animals were restricted to the IC_contra_ in response to BBN stimuli. Thus, it is conceivable that mild residual hearing loss in tinnitus animals may have further exacerbated this reduction in perceived sound level, thereby resulting in the observed significant difference between controls and tinnitus groups only in response to BBN stimuli. Nevertheless, it should be noted that – in the same neurons – gap detection in response to pure tones remained largely intact. Furthermore, 50 ms gaps were detectable by the majority of IC neurons, regardless of the stimulus presented, and gaps of 1 ms duration were still detectable by a similar number of neurons in controls, no tinnitus, and tinnitus GPs.

Another limitation of the present study was that a number of units either did not respond strongly to any of the frequencies in the NBN condition or their CFs were not within 1 kHz of the frequency range of the NBN, hence an MGDT could not be reliably obtained. Consequently, the sample size for this condition is substantially lower than pure tone or BBN conditions. Nonetheless, the average MGDT for units that were responsive to NBN was considerably <50 ms gap duration of the behavioral test. Furthermore, it has previously been shown that psychophysical GDTs may feasibly be determined by across-channel integration of responsive neurons (44). Given that pure tones (at the CF of a cell) generate the most robust sound-evoked response, it can be assumed that the mean MGDT to the pure tone gap condition is a reasonable predictor of behavioral GDTs. The fact that pure tone responses had, on average, similar MGDTs in tinnitus, no tinnitus, and control animals, suggests that gross deficits in neural gap detection were not responsible for the behavioral gap detection deficits observed in the same animals.

It is also important to note that gaps of different durations are employed by other researchers demonstrating behavioral gap detection deficits, including 75 ms (45), 40 ms (34), 20 ms (11), and 15 ms (10, 26). However, in the present study, a similar percentage of neurons had MGDTs of ≤10 ms in response to pure tones in tinnitus GPs, compared with controls and no tinnitus GPs (80 vs. 77 and 74%, respectively). Furthermore, a large number of units were still able to respond to gaps of 1 ms duration following AOE. Therefore, it is unlikely that temporal processing deficits following AOE were causing behavioral gap detection deficits, even in studies employing gaps of shorter durations.

Fournier and Hebert (14) suggested that tinnitus may not be filling in the gap *per se*, as the deficits in gap detection they observed in patients with high-frequency tinnitus (by measuring the eye-blink reflex) were not limited to a high-frequency background carrier, but were also present in a low-frequency carrier condition. However, the frequency most similar to patients’ tinnitus (16 kHz), as determined using a likeness-matching procedure [e.g., Ref. (46)], was not matched to the frequency of the background carrier (4 kHz for the high-frequency condition; 500 Hz for the low-frequency condition). Consequently, it would have been of significant interest to determine whether gap detection deficits were augmented at the tinnitus frequency compared with the other frequencies tested. Contrary to the results of Fournier and Hebert (14); Campolo et al. (47) demonstrated that people with tinnitus were still capable of psychophysical gap detection when the gap background carrier was matched to tinnitus frequency. It is possible, however, that fundamental differences in the paradigms (e.g., using a reflex response compared with a cognitive task) may explain these seemingly contradictory results. Further studies are needed that use carrier frequencies more similar to the characteristics of patients’ tinnitus, as well as comparing the two different tasks (reflexive and cognitive), in order to determine whether the hypothesis of “filling in” the gap is applicable to human beings.

In the present study, we did not measure the spontaneous firing rates of IC neurons. Although elevated spontaneous firing has previously been demonstrated following AOE [e.g., Ref. (18, 19)], we recently established that this was not solely indicative of tinnitus in our model owing to the fact that elevated spontaneous firing was also present in AOE-treated animals with no behavioral evidence of tinnitus (8). Nevertheless, it is plausible that increased spontaneous firing at a cortical or subcortical level could render the gap undetectable, thus, causing a reduction in temporal acuity, an idea proposed previously (48). If this was indeed the cause of impaired behavioral gap detection, it would be anticipated that gap detection is significantly impaired in IC neurons. Given that only slight changes in neural GDTs were observed in tinnitus animals compared with both unexposed controls and no tinnitus GPs, it therefore seems unlikely that elevated spontaneous firing at the level of the IC is the direct cause of impaired behavioral gap detection. However, increased spontaneous activity at the level of the auditory cortex cannot be discounted as a contributing factor to impaired behavioral performance.

A right-side advantage in temporal processing was identified for unexposed controls, no tinnitus GPs, and tinnitus GPs in response to pure tones, i.e., mean MGDTs in IC_ipsi_ were considerably longer than IC_contra_. However, the sample sizes in these data were relatively small and definitive conclusions cannot therefore be drawn. In human beings, some psychophysical studies have previously reported that a left hemisphere advantage was evident in temporal processing [e.g., Ref. (49, 50)], contrasting with the right-side advantage demonstrated here. However, other psychophysical studies failed to reproduce this left hemisphere advantage in human beings [e.g., Ref. (51, 52)]. Nonetheless, the data presented here conflict with human psychophysical literature reporting a left-side advantage. Furthermore, Wetzel et al. (53) found that, in gerbils, the detection of gaps in frequency-modulated tones in a behavioral task was impaired by left, but not right, auditory cortex lesions, suggesting hemispheric differences in temporal processing in this species. It is highly plausible that differences between these studies and the paradigm used here may play a role in any inconsistencies in these findings (e.g., species differences, the use of anesthetics, or procedural differences, i.e., electrophysiology vs. psychophysics). Interestingly, fMRI studies in human beings support the findings of a right-side advantage and contradict the human psychophysical results, demonstrating increased activation in the right hemisphere that was better-correlated with performance on a temporal processing task than was the case for the left hemisphere (54, 55). Further investigation of hemispheric temporal processing differences in animals is necessary to address these discrepancies in the literature.

It was particularly intriguing that there was a significant increase in the proportion of units exhibiting onset responses following AOE. The proportion of onset/sustained responses in control animals shown here was very similar to that reported previously for urethane-anesthetized GPs [21% onset vs. 73% sustained; (56)]. Changes in response types of units recorded in the IC have previously been demonstrated following bicuculline and strychnine administration (56). For example, ~50% of units demonstrated changes in PSTHs following application of either drug, most commonly transforming into “chopper” responses, characterized by a regular discharge pattern of three or more peaks near the stimulus onset, regardless of the previous unit response classification. These changes in response type were attributed to the antagonistic effect that these drugs have on GABA and glycine receptors. As an added confound, the class of anesthetic can affect the proportion of different temporal response types, the percentage of onset units recorded in the IC of control GPs was significantly greater under pentobarbital anesthesia than ketamine or urethane (31). In contrast to the data presented here, studies in the GP (57) and the chinchilla (58) showed no marked change in the types of single-unit responses in the IC after AOE. However, these findings related to recordings performed immediately after acute noise exposure, whereas the data presented here provide the first evidence for long-term changes in response types following AOE.

There is evidence indicating that IC units with different types of responses may have different functional roles. For example, Zheng and Escabi (59) demonstrated that sustained units are effective at encoding the envelope shape of stimuli with low-modulation rates, while onset units are most suited to representing repetitive stimuli at high-modulation rates. The response properties of IC units are largely determined by inhibitory inputs (56), mediated by GABA and glycine inhibitory neurotransmitters. Wallace et al. (60) suggested that onset responses were likely to come from multipolar cells rather than from laminar cells in the IC, while sustained units were recorded from both flat laminar cells and multipolar cells, highlighting possible morphological differences underlying the two response types. It seems unlikely that AOE would cause a complete change in morphological class, i.e., from laminar to multipolar cell, to account for the change in the proportion of onset neurons. However, multipolar cells previously exhibiting sustained responses could be altered to produce onset responses. As yet, it is unclear how this change may be caused by AOE. Changes in the balance of excitation and inhibition, as observed following acoustic trauma and linked to tinnitus (61–63), could possibly contribute to the changes in the response patterns of units observed here. However, given that inhibitory antagonists (such as bicuculline or strychnine) increased the discharge rate of neurons in the IC (56), the shift toward a higher proportion of onset cells following AOE observed here, wherein inhibition is likely to be reduced [for a review, see Ref. (64)], appears to be counterintuitive.

At present, given the current evidence, a gross change in neural GDTs within the IC is unlikely to be the basis of behavioral gap detection deficits. Furthermore, the original perceptual “filling in” hypothesis presented by Turner et al. (6) presumably involves the forebrain (65, 66). Previous work in rats established that auditory cortex ablation increased behavioral GDTs (67); thus, the auditory cortex may be a candidate for involvement in the increased neural thresholds shown here. The auditory cortex has a profound effect on temporal processing in the IC (68), and it is possible that corticofugal modulation might be central in changing the proportion of onset cells in the IC. Further research relating changes in response types to tinnitus may prove useful in elucidating the mechanisms by which the IC contributes to the generation of the tinnitus percept.

## Conflict of Interest Statement

The authors declare that the research was conducted in the absence of any commercial or financial relationships that could be construed as a potential conflict of interest.
